# Bronchopleural Fistula in Tuberculosis: A Challenge to Mechanical Ventilation

**DOI:** 10.7759/cureus.76756

**Published:** 2025-01-01

**Authors:** Diogo Alves, Rogério Corga da Silva, Rita Morais Passos, Joana Abreu, José Sá

**Affiliations:** 1 Department of Critical Care Medicine, Unidade Local de Saude do Alto Minho, Viana do Castelo, PRT

**Keywords:** antituberculosis therapy, bronchopleural fistula, critical care, mechanical ventilation, pulmonary complications, single lung ventilation, tuberculosis

## Abstract

Pulmonary tuberculosis (TB) remains a significant cause of morbidity and mortality worldwide, particularly in resource-limited settings. Bronchopleural fistula (BPF) is a rare yet life-threatening complication of TB, presenting substantial diagnostic and therapeutic challenges. Its effective management demands a multidisciplinary approach, especially when compounded by concurrent infections and severe malnutrition. We present the case of a 39-year-old male with a history of chronic alcohol use and heavy smoking, who presented with severe cachexia, pneumothorax, and a persistent air leak. Diagnostic evaluations confirmed TB complicated by BPF and superinfection with *Streptococcus pneumoniae*. Despite initiating antitubercular therapy, antibiotics, and thoracic drainage, the patient’s condition deteriorated rapidly. Given the high surgical risk, conservative measures, including single-lung ventilation and supportive care, were employed. However, his clinical course was marked by progressive respiratory distress, refractory septic shock, and multiorgan failure. Despite exhaustive supportive efforts, the patient succumbed to the illness on the eighth day of hospitalization. This case underscores the complexities of managing TB-associated BPF, particularly in patients with severe malnutrition and coexisting infections. It highlights the critical need for early diagnosis, individualized interventions, and close multidisciplinary collaboration to optimize outcomes in such challenging scenarios.

## Introduction

Pulmonary tuberculosis (TB) remains a significant global health challenge, particularly in low- and middle-income countries where its burden is highest. WHO, as the leading authority on international health within the United Nations system, plays a pivotal role in combating TB. According to WHO’s 2024 report, TB is a preventable and often curable disease. However, in 2023, it regained its position as the world’s leading cause of death from a single infectious agent, surpassing COVID-19 after three years, and causing nearly twice as many deaths as HIV/AIDS. Over 10 million people contract TB annually, with case numbers increasing since 2021, although the global incidence began stabilizing in 2024. Early diagnosis and adherence to WHO-recommended treatments - a four- to six-month course of anti-TB drugs - can achieve a cure rate of approximately 85%, underscoring the importance of timely and accessible treatment [1].

Bronchopleural fistula (BPF) is a relatively rare but life-threatening complication of TB [2]. Defined as a pathological connection between the pleural cavity and bronchus with a persistent air leak, the term “BPF” is often used interchangeably with alveolar pleural fistula, which refers to air leaks originating from the lung parenchyma. The duration of air leaks defining BPF varies in the literature, ranging from 24 hours to five days, although leaks lasting over 24 hours are commonly accepted as BPF [3].

BPF most frequently arises as a postoperative complication of pulmonary resection, particularly pneumonectomy. Other etiologies include lung necrosis from infections, persistent spontaneous pneumothorax (PSP), chemoradiotherapy, and TB [4]. Despite its rarity, BPF substantially complicates clinical management and is associated with high mortality rates, reaching up to 67% in severe cases [5,6].

In TB patients, BPF typically results from the rupture of pulmonary cavities into the pleural space or the erosion of pleural empyema into the lung. Its clinical manifestations are diverse, ranging from incidental air-fluid levels on chest radiographs to life-threatening tension pneumothorax [2]. These complications are often exacerbated by the immunosuppression and malnutrition commonly associated with chronic TB, further worsening patient outcomes [4,7,8].

BPF disrupts normal pleural pressure dynamics, leading to PSP and impaired pulmonary function [3,9]. Managing this condition is challenging. While surgical intervention remains the definitive treatment in many cases, conservative strategies, such as mechanical ventilation techniques - including one-lung ventilation - are employed to stabilize patients or bridge them to recovery [10-14].

This case report describes a rare and complex presentation of pulmonary TB, complicated by BPF and PSP, in a patient who also developed an associated pneumonia.

## Case presentation

A 39-year-old male with a history of heavy smoking (40 pack-years) and chronic alcohol use presented to the emergency department with complaints of acute worsening of chronic shortness of breath and cough, which had progressively worsened since the previous day. He reported a six-month history of significant weight loss (33% of total body weight, from 65 kg to 44 kg), along with two months of night sweats and low-grade fever. His medical history included a family history of TB, with his father having died of the disease several years earlier. Despite this, the patient had previously refused TB screening.

On physical examination, the patient exhibited severe cachexia and cyanosis, with tachycardia measured at 120 beats per minute, and no hypotension. He was tachypneic (respiratory rate: 30 cycles per minute), and his oxygen saturation (SpO₂) was unmeasurable on room air. Blood gas analysis revealed hypoxemia (pO₂: 63 mmHg), hypocapnia (pCO₂: 24 mmHg), elevated lactate levels (3.9 mmol/L), normal bicarbonate levels (cHCO3: 22 mmol/L), and alkalemia (pH: 7.48). Initial blood work demonstrated an elevated CRP level (16.90 mg/L), indicating significant inflammation, while the white blood cell count was within the normal range (7.560 × 10³/µL) (Table 1).

**Table 1:** Analytical results at admission and on subsequent days FiO₂, fraction of inspired oxygen; HFNC, high flow nasal cannula; pCO₂, partial pressure of carbon dioxide; pO₂, partial pressure of oxygen; SLV, single-lung ventilation

Parameter	Emergency department	Day of ICU admission	After initiating SLV	Reference range
FiO₂ and method of delivery	Breathing room air	FiO₂: 60%, HFNC	FiO₂: 60%, mechanical ventilation	
Total WBC count (× 10³/µl)	7.56	4.75	5.99	4.000-10.000
CRP (mg/dL)	16.9	13.72	7.22	<0.51
Arterial blood gas				
pH	7.48	7.24	7.29	7.35-7.45
pO₂ (mmHg)	63	58	105	80-100
pCO₂ (mmHg)	24	75	81	35-45
Bicarbonate (mmol/L)	22	26.7	38.9	22-26
Lactate (mmol/L)	3.9	3.4	1.2	<1.9

**Figure 1:**
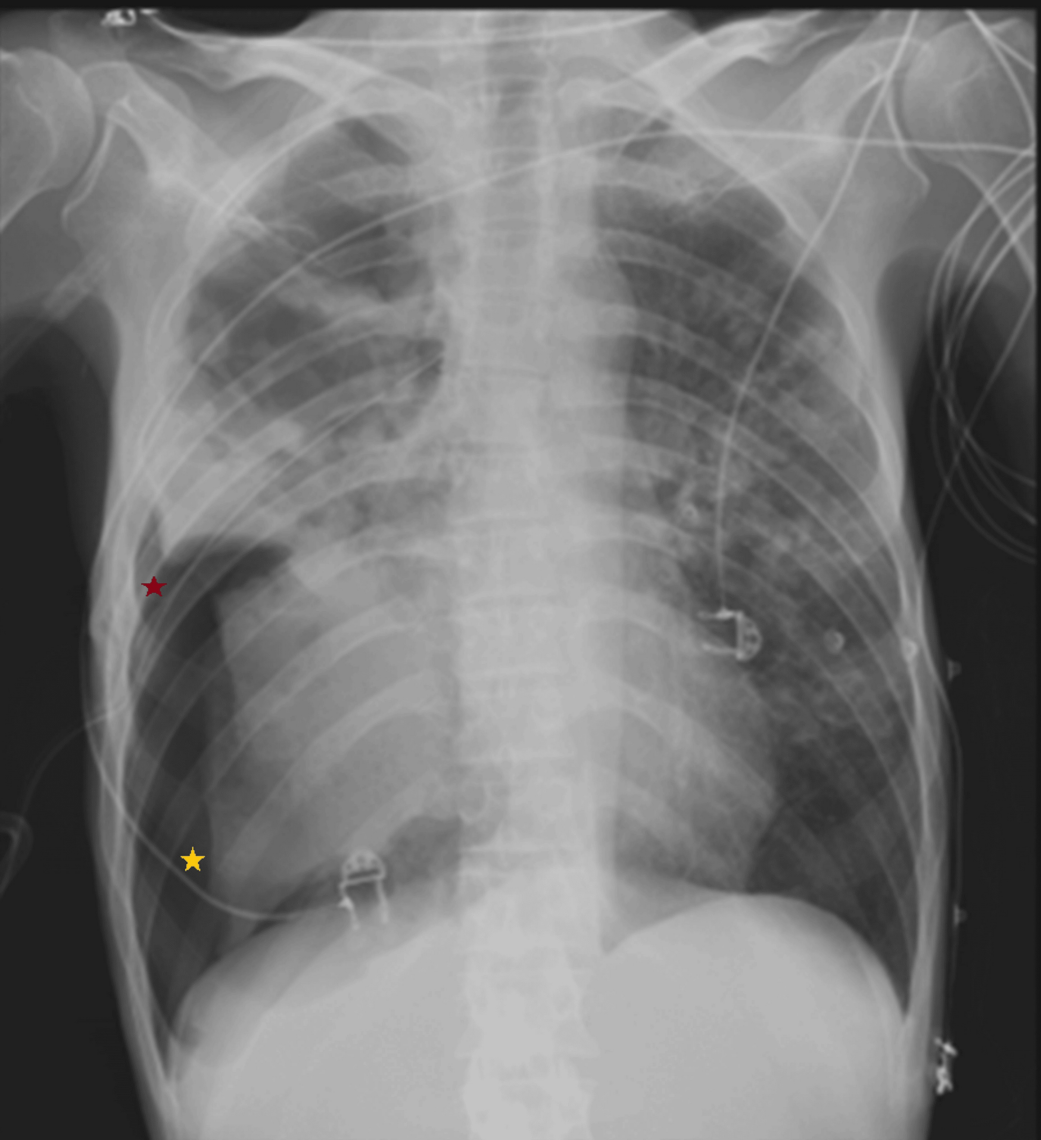
Chest radiograph following chest tube placement Emergency department chest radiograph after chest tube placement (red star), showing extensive bilateral lung opacities and a persistent right-sided pneumothorax (yellow star).

Chest X-ray revealed a large right-sided pneumothorax and diffuse left-sided opacities. An 18 French chest tube was inserted in the fifth intercostal space in the midaxillary line, using a blunt dissection technique (Figure 1), and connected to an underwater seal drainage system with visible bubbling. The patient experienced partial symptom relief, but continued to require oxygen therapy to maintain SpO₂ above 92%.

**Figure 2:**
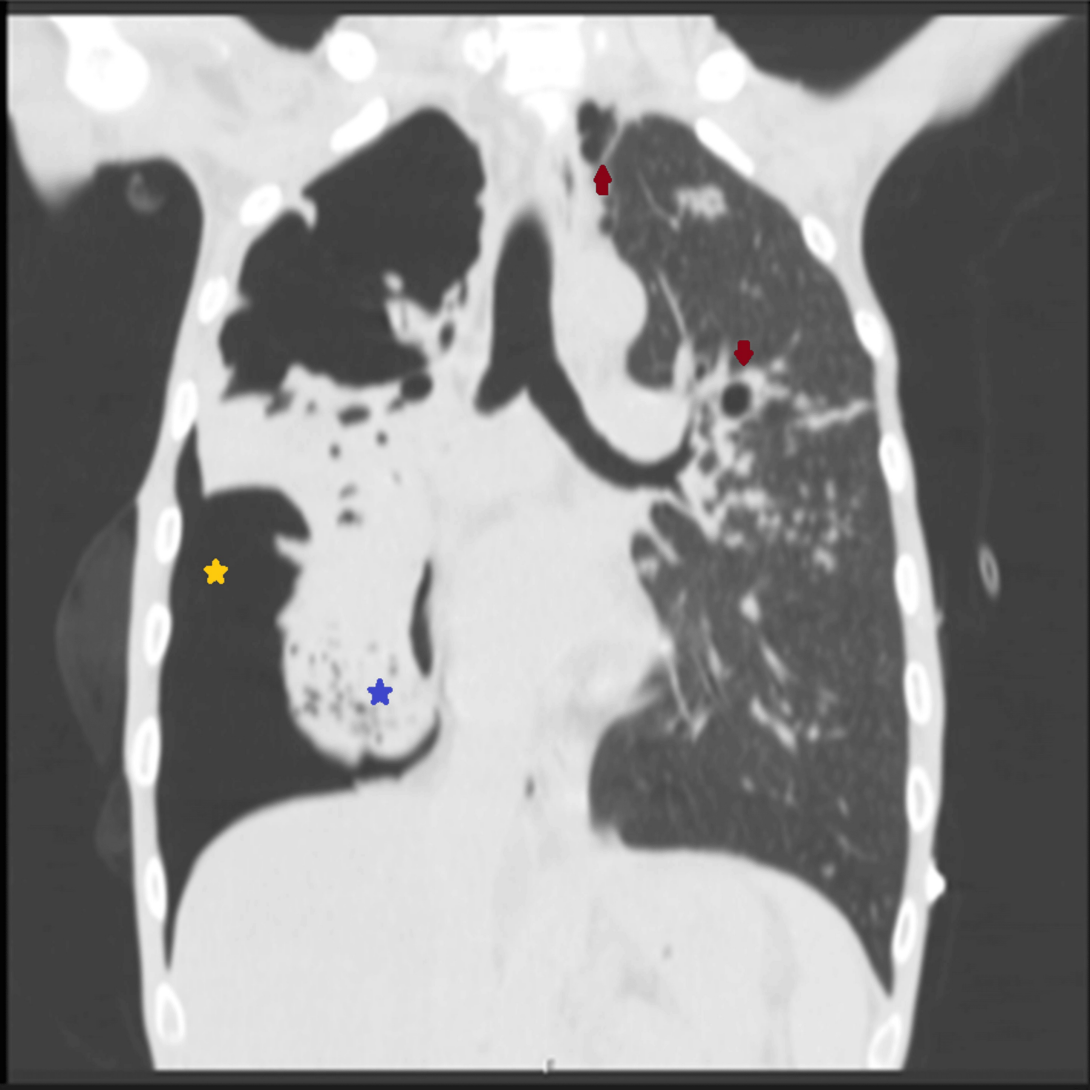
Coronal chest CT scan image Coronal image showing an extensive right-sided pneumothorax (yellow star) with lung atelectasis (blue star) and small cavitations visible in the left lung (red arrows).

**Figure 3:**
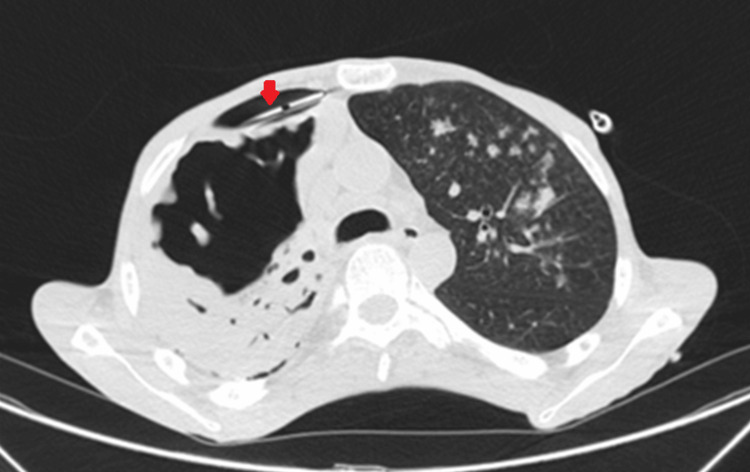
Axial chest CT scan image Axial image showing correct chest tube placement (red arrow) with persistent pneumothorax.

Following this, a CT scan of the chest revealed multiple peribronchovascular nodules in the left lung, predominantly in the upper lobes, with some cavitated lesions, the largest measuring 13 mm (Figure 2, red arrows). A large-volume pneumothorax (Figure 2, yellow star) was observed on the right side, accompanied by a small-volume pleural effusion and near-complete atelectasis of the right lung (Figure 2, blue star). Additionally, a cavitated lesion with a multilobulated nodular wall was noted in the right upper lobe, consistent with TB. The chest tube was confirmed to be well-positioned (Figure 3, red arrow) and still bubbling, raising suspicion for the presence of a BPF.

Microbiological testing performed at admission revealed a positive *Streptococcus pneumoniae* antigen in the urine. In addition, the cartridge-based nucleic acid amplification test of sputum was positive for *Mycobacterium tuberculosis*, which was sensitive to rifampicin. These findings confirmed a diagnosis of pulmonary TB with superinfection by *S. pneumoniae*. Screening for HIV coinfection, as well as an expanded PCR panel for respiratory viruses, including COVID-19 and influenza, were negative.

Based on these results, the patient was started on anti-TB therapy (isoniazid, rifampin, pyrazinamide, and ethambutol), alongside amoxicillin-clavulanate for the *S. pneumoniae* superinfection. Despite these interventions, the patient's condition continued to decline, with worsening respiratory distress and hypoxia. The digital chest drain system revealed persistent air leakage exceeding 5 liters per minute. On day 2 of hospitalization, the patient was transferred to the ICU and started on high-flow nasal cannula therapy in an attempt to correct hypoxia without using positive pressure ventilation. Thoracic surgery was consulted regarding the management of the BPF, and it was collectively decided to adopt a conservative approach. The pneumothorax was thought to have predated the patient's acute decompensation, which was attributed to superinfection with *S. pneumoniae*. The plan was to maintain pleural drainage using the chest tube, manage the infection and sepsis, and continue anti-TB therapy. Definitive surgical intervention, such as pneumonectomy, was deferred until the superinfection and TB were better controlled.

Consultation with an extracorporeal membrane oxygenation (ECMO) team was also performed to determine whether the patient could benefit from veno-venous ECMO, which would allow minimal lung ventilation to facilitate the healing of the BPF. However, the patient was deemed unsuitable for extracorporeal support due to his severe cachexia and extensive bilateral lung cavitation.

**Figure 4:**
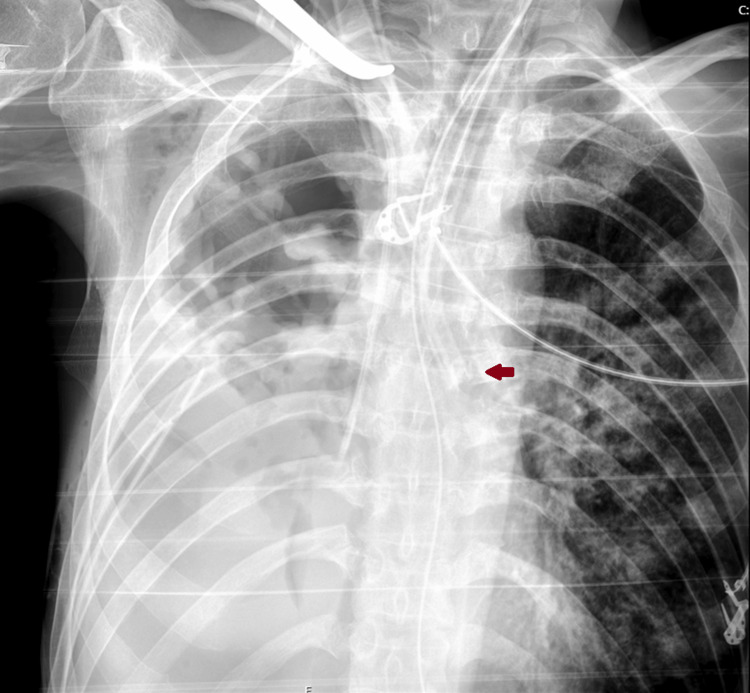
Chest radiograph during SLV Chest radiograph showing correct placement of a left-sided double-lumen tube (red arrow). SLV, single-lung ventilation

Following the decision to proceed with a conservative approach and after the failure of noninvasive methods to correct hypoxia, the patient was intubated with a 35 French double-lumen endotracheal tube (DLET) and placed on single-lung ventilation (SLV) of the left lung to manage the significant air leak and respiratory compromise (Figure 4). This strategy successfully reduced the air leak to less than 100 mL/hour. However, ventilation of the left lung remained suboptimal due to extensive scarring, cavitations, and pneumonia. Ventilatory optimization was limited, and a permissive hypercapnia strategy was adopted, utilizing low positive end-expiratory pressure (PEEP) of 2-5 cmH₂O, tidal volumes of approximately 4-6 mL/kg of ideal body weight, and a high respiratory rate of 28-32 cycles per minute, resulting in a pCO₂ of approximately 80-110 mmHg and a tolerated mild acidemia with a pH of 7.25-7.35 (Table 1).

Over the next few days, the patient’s condition continued to deteriorate, with worsening septic shock despite receiving broad-spectrum antibiotic therapy and a downtrend in CRP levels. The first sputum cultures collected at hospital admission were inconclusive, as multiple bacterial isolates were identified. A flexible bronchoscopy was attempted during the ICU stay, but the small diameter of the DLET and the lack of pediatric fibroscopes at our hospital prevented the procedure. A blind endotracheal aspirate from the left side of the DLET later yielded negative results. Pneumonia showed no response to therapy and progressed, with worsening infiltrates in the left lung on chest X-ray. The ventilated left lung became increasingly noncompliant, leading to significant challenges in maintaining adequate ventilation. This decline in pulmonary function resulted in worsening respiratory acidosis, which progressed to acidemia despite ventilatory optimization.

As the left lung’s compliance continued to decrease, the team faced escalating difficulty in ventilating the patient effectively. The combination of refractory septic shock and progressive respiratory acidosis significantly worsened the patient’s prognosis.

Despite supportive measures, including high-dose vasopressor support with noradrenaline up to 2.5 µg/kg/min and vasopressin at 0.03 IU/min, the patient evolved to refractory respiratory acidosis (pCO₂ >120 mmHg), acidemia (pH <7.1), and cardiovascular collapse, succumbing to multiorgan failure on day 8 of hospitalization.

## Discussion

The initial management of tuberculous BPF typically involves tube drainage and antibiotic therapy. While tube thoracostomy is widely used as a temporary measure to rapidly resolve sepsis and prepare patients for more definitive therapy, once pleural drainage is established, sepsis is controlled, and TB is adequately treated, low-risk patients can be considered for permanent fistula repair. Preparation for surgery typically takes 3-6 months to minimize complications such as pleural empyema or bronchial stump dehiscence. Optimal nutrition and antituberculous therapy are essential. Definitive surgery aims to close the fistula, re-expand the lung via decortication if necessary, and fill the residual space with re-expanded lung tissue, vascularized muscle, or omental flaps. Pulmonary resection is reserved for irreparably damaged lung tissue [2,15].

Not only is BPF a high-mortality complication of TB, but it also poses significant risks for mechanically ventilated patients. A review of 1,700 mechanically ventilated patients found that 2% had BPF secondary to barotrauma or direct lung injury, with an overall mortality of 67% [3].

There is a paucity of data and no established consensus or guidelines for managing BPF. Ventilating patients with BPF presents significant challenges due to the interaction between the fistula and the mechanical ventilation process. BPF creates a direct communication between the bronchial tree and the pleural space, causing a persistent air leak that undermines effective ventilation. PEEP maintains positive pressure at the airway opening at the end of expiration, distending distal alveoli, increasing functional residual capacity, and improving lung compliance. While PEEP enhances oxygenation, the air leak caused by BPF leads to loss of PEEP, compromising alveolar recruitment and resulting in hypoxemia. Additionally, BPF reduces tidal volume due to low resistance to flow, exacerbating ventilation-perfusion mismatch and hindering adequate gas exchange [3,16,17].

Managing these issues is complex because increasing ventilatory pressures to improve oxygenation can inadvertently enlarge the fistula or create new ones, thereby exacerbating the air leak and perpetuating a cycle of ventilatory insufficiency. For example, higher peak inspiratory pressures and PEEP levels, although intended to support oxygenation, may inadvertently amplify the air leak by increasing mean airway pressure and delaying healing, as shown in both animal models and clinical observations [3,16].

SLV has emerged as a critical tool in managing BPF by isolating the diseased lung to reduce airflow through the fistula, thereby promoting healing while maintaining oxygenation. SLV, achieved using DLETs or bronchial blockers, minimizes pressure and volume changes in the affected lung, preventing further air leakage and allowing the contralateral lung to maintain adequate gas exchange [3,9-12].

Minhas et al. demonstrated that SLV could improve oxygenation and facilitate fistula closure in cases of BPF complicating acute respiratory distress syndrome [10]. Similarly, Cheatham and Promes reported its successful application in a trauma patient with high-output BPF, showing significant improvement in pulmonary compliance and resolution of the fistula [11].

In TB-associated BPF, SLV offers additional benefits, such as reducing the risk of cross-contamination between the diseased and healthy lungs and optimizing ventilatory pressures to mitigate air leaks [3,12]. However, SLV requires careful ventilatory management to avoid complications like hypoxemia and hypercapnia in the ventilated lung [4,12,18]. Despite these challenges, SLV has proven effective as a bridge to recovery or surgical intervention in refractory cases [8,11].

In the presented case, the patient did not respond to the administered antibiotic therapy, and sepsis could not be controlled. Despite consultation with the thoracic surgery team, a conservative approach was chosen due to the high surgical risk in the initial treatment phase.

Video-assisted thoracoscopic surgery (VATS) is increasingly used for managing BPF, offering advantages typical of minimally invasive surgery, such as reduced postoperative pain, shorter hospital stays, and faster recovery times compared to open thoracotomy [19]. The enhanced visualization provided by VATS allows precise dissection and secure fixation of vascularized grafts, essential for effective fistula closure. However, definitive treatment should be delayed until the infection is under control and the patient’s nutritional status is optimized [20].

Flexible bronchoscopy, traditionally used for diagnostic purposes, has also gained traction as a therapeutic modality for managing BPFs, particularly central BPFs, often following lobectomy and sometimes after pneumonectomy. The technique aims to reduce life-threatening air leaks, prevent aspiration of pleural fluid, and mitigate secondary pleural contamination, especially in fragile patients who are unfit for immediate surgical intervention. While flexible bronchoscopy has shown promise as a temporary measure, it is primarily indicated for small fistulas and should not be considered a definitive treatment. Its role is to stabilize the patient and improve clinical conditions, facilitating subsequent definitive surgical repair when feasible [20].

The fatal outcome in this case was influenced by multiple factors, including delayed presentation despite prolonged TB symptoms, superinfection with *S. pneumoniae*, severe malnutrition, and the need for prolonged SLV, which added strain on the already diseased left lung. These factors contributed to refractory acidosis and septic shock, which ultimately proved fatal. Earlier diagnosis, aggressive nutritional support, and advanced interventions such as ECMO, although not feasible in this case, might have improved the outcome.

## Conclusions

This case highlights the complexity of managing BPF as a severe complication of TB, especially in patients with significant comorbidities such as malnutrition and secondary infections. The interaction between critical illness, persistent air leaks, and the challenges of mechanical ventilation underscores the need for a multidisciplinary approach. The patient's poor outcome emphasizes the importance of early detection, aggressive nutritional and medical optimization, and tailored interventions to stabilize critically ill patients before pursuing definitive surgical repair. It also stresses the need for further research to develop standardized guidelines for managing TB-associated BPF, including innovative ventilation strategies and alternative therapies. Finally, this case underscores the urgency of strengthening public health efforts to address TB as a preventable and curable disease, with improved access to early screening and treatment to minimize life-threatening complications.
